# Social Networks, Cultural Pride, and Historical Loss Among Non-Reservation American Indian / Alaska Native Emerging Adults

**DOI:** 10.21203/rs.3.rs-3547685/v1

**Published:** 2023-11-20

**Authors:** David P. Kennedy, Ryan A. Brown, Elizabeth J. D’Amico, Daniel L. Dickerson, Carrie L. Johnson, Nipher Malika, Anthony Rodriguez, Virginia Arvizu-Sanchez

**Affiliations:** RAND Corporation; RAND Corporation; RAND Corporation; University of California, Los Angeles; Sacred Path Indigenous Wellness Center; RAND Corporation; RAND Corporation; Sacred Path Indigenous Wellness Center

## Abstract

Health disparities among American Indian/Alaska Native (AI/AN) populations in the United States are the result of historical traumas, such as colonization, forced relocation, and federal policies focused on cultural assimilation. Culturally-tailored health interventions aim to address intergenerational trauma by emphasizing cultural strengths and building positive social connections. In this article, we explore the social network characteristics of participants of the first culturally-tailored health intervention for AI/AN emerging adults (18–25) living outside of tribal lands. Participants (N = 150; 86% female) were recruited across the United States via social media and completed online egocentric network interviews prior to the start of intervention workshops. Participants’ networks were diverse in composition and structure. They were primarily composed of family and friends, were people they had regular contact with, were similar age, and provided participants with support. We tested for significant associations between network characteristics, individual characteristics (age, gender, travel to reservations, speaking tribal languages, etc.) and two dependent measures: 1) cultural pride and belongingness and 2) thoughts of historical loss. Multiple regression results show that higher proportions of network members who discussed AI/AN identity with participants and having more network members who engage in traditional practices was associated with stronger cultural pride and belongingness. Higher proportions of network members having discussion of AI/AN identity with participants was also associated with more frequent thoughts of historical loss. Controlling for network factors, no individual characteristics were associated with either dependent variable. We discuss implications for the development of culturally-tailored health interventions.

## Introduction

American Indian/Alaska Native (AI/AN) people in the United States (U.S.) experience a disproportionate rate of morbidity and mortality compared to other Americans. AI/AN people have one of the lowest life expectancies compared to other racial/ethnic groups in the U.S. and the gap between AI/AN and White populations has increased in recent years [1]. Mortality among AI/AN people is tied to disproportionately high rates of many interconnected negative health conditions. For example, AI/AN people have the highest rate of diabetes in the nation, with a prevalence (14.7% in 2018) of more than twice that of non-Hispanic White people [2]. AI/AN adults are also more likely to be obese than Non-Hispanic White adults [3, 4]. Prevalence rates for drug and alcohol use [5] and smoking [6] are higher for AI/AN people compared to US overall population rates. AI/AN people in non-reservation areas, where currently more than 87% of AI/AN people reside [7], are significantly more likely to die from heart disease, cancer, unintentional injury, diabetes, and chronic liver disease and cirrhosis compared to White people living in urban areas [8]. AI/AN people are also disproportionately affected by suicide compared to the general U.S. population [9, 10].

A disproportionate experience with trauma and post-traumatic stress appears to play a key role in the morbidity and mortality disparities experienced by AI/AN populations [5, 11, 12]. For example, generations of AI/AN people in the U.S were subjected to forced relocation from traditional tribal lands to designated U.S. cities, due in part to the Relocation Act of 1956 [13], which resulted in a large number of AI individuals who moved to urban areas becoming unemployed, homeless, and disconnected from their community-based support networks [14, 15]. Studies have found significant links between experience of trauma and substance use [16] and suicide [5, 12, 17] among AI/AN people. Elevated rates trauma exposure include both direct experiences, such as childhood abuse or neglect [18], and indirect experiences through shared cultural, historical, and inter-generational trauma [19].

The resulting shared experience of trauma and stress has been directly and indirectly linked to significant health disparities among AI/AN populations [20, 21]. Prevalent and persistent conditions of poverty and social disconnection and isolation have contributed to continued, inter-generational health-related disparities for urban AI/AN people [14, 15]. Direct experiences of traumatic cultural loss, such as attendance of boarding schools, have also been found to be associated with higher rates of drug and alcohol use and suicide ideation [22]. Forced relocation has also been found to have a ripple effect on subsequent generations’ negative alcohol experiences and depression [23]. Recurring thoughts of the past generations’ experiences of historical loss are associated with suicide ideation [12, 17, 22, 24], behavioral addiction [16, 25], and depression [26–29].

To counter the inter-generational experience of trauma and social fragmentation, many recent interventions have been developed to improve the health of AI/AN populations by promoting positive concepts of AI/AN identity, encouraging engagement in traditional practices and increased community involvement, and improving social connections among AI/AN people [30–42]. Health interventions have also been culturally tailored for AI/AN people living in urban areas [43–47]. These programs focus on increasing overall well-being and healthy behaviors by addressing stress linked with cultural identity, stigma, and community connections - as well as providing ways to promote health through engagement in traditional practices and culture – which is often is often protective and associated with decreased risk behavior [37–40, 43, 44, 48–50]. In addition to promoting cultural strengths, some programs also explicitly aim to reduce the negative effects of historical trauma for Indigenous populations [51, 52].

Some researchers have called for expansion of AI/AN culturally-tailored interventions to include an emphasis on social networks and the role of social relationships in health [31]. An important aspect of many interventions that promote cultural strength and cultural pride is building a sense of social connectedness with other people who share the same AI/AN identity. Although ethnic identity is often assumed to be synonymous with demographic and/or biological characteristics, it is multi-faceted and strongly influenced by social factors [53]. Ethnic identity, similar to many other personal characteristics and behaviors, is shaped by existing social relationships [54] through two well established social network dynamics, network selection and influence [55]. Studies of adolescent development have found that peer networks are a key driver of the development of ethnic identity [56–58]. The social context of ethnic identity is especially important for interventions that address health disparities among AI/AN adolescents and emerging adults who do not live on tribal lands / reservations (hereafter referred to as “urban” areas). Urban AI/AN adolescents and emerging adults may be socially isolated from other AI/AN peers as well as from family members and tribal communities living in rural tribal lands/reservations. Limited connections to people who share AI/AN identity coupled with extensive interactions and influences from non-AI/AN people in urban areas is a potential barrier to promoting positive concepts of AI/AN identity, engagement in traditional practices, and increased involvement in AI/AN community activities for adolescents and emerging adults living in urban areas. This challenge is heightened for emerging adults, who are also developing new social roles and expanding their social networks beyond their families and school-based friendships.

One barrier to developing culturally-tailored health interventions for AI/AN emerging adults living in urban areas is the lack of research on their social networks. To date, social network research on AI/AN emerging adults is sparse, despite decades of findings in other populations linking social factors with health [59], including major drivers of health disparities among AI/AN populations, such as substance use [60–62]. Very little research with AI/AN communities in general - and no research with AI/AN emerging adults in particular - has addressed the importance of social networks in supporting well-being. Some studies have shown a protective influence of network ties in discouraging behaviors that have negative health outcomes among urban AI/AN youth ages 9 to 17 [62]. Other studies have investigated the role of social networks among AI/AN teens living in tribal areas and in relation to suicide attempts and prevention [62–64]. To date, there have been no studies of the social networks of AI/AN emerging adults who primarily live in urban areas.

## Current Study

This study addresses these gaps by presenting empirical findings about the social networks of urban AI/AN emerging adults. These findings can help inform the development of culturally-tailored health interventions to address health disparities resulting from historical, inter-generational, and ongoing trauma and discrimination. Our first aim is to describe the social networks of 150 urban AI/AN emerging adults who were recruited for a randomized control trial (RCT) to test the efficacy of a workshop-based, culturally-tailored substance use prevention intervention for urban AI/AN emerging adults [65]. We present results from a baseline personal network survey [66, 67] of recruited participants prior to the intervention sessions. We examine types of people in their network (e.g., friends, family, co-workers), the age of social network members, the geographic distance from network members, how frequently they interact with network members, and how many people in their personal networks provide support to participants. We also examine the percent of network members who identify as AI/AN, engage in traditional practices, and discuss AI/AN identity with our survey participants. We also analyze the structure of the participants’ networks, specifically how interconnected the members of these networks are to each other. Our second aim is to test for associations between social network characteristics and two key components of many culturally-tailored health interventions for AI/AN people: 1) cultural pride and belonging and 2) thoughts of historical loss. Finally, our third aim is to test for associations between individual characteristics of participants and cultural pride and belonging and thoughts of historical loss. The individual characteristics we examine include age, sex/gender minority identity, socio-economic status, if participants were born on a reservation/tribal land, how much of their lives they have lived in urban vs. tribal/reservation lands, and if they speak a tribal language.

First, in describing social networks, we expect that AI/AN emerging adults living outside of reservations or tribal lands will have many types of people in their lives, including a mixture of AI/AN and non-AI/AN connections. Second, we examine associations between these social network characteristics and participants’ (a) internal feelings of cultural connectiveness, as well as (b) their thoughts regarding historical loss using regression models. We expect that urban AI/AN emerging adults who have more network members who report AI/AN identity, who engage in traditional practices, who they regularly discuss their AI/AN heritage, and who have experienced living on reservations/tribal lands will have stronger feelings of connectivity to their cultural heritage and will also be more preoccupied with thoughts about the losses of culture, land, and people experienced by generations of AI/AN people as a result of colonization. We also expect that structural characteristics of personal networks will be associated with feelings of cultural connection. Personal networks with higher density are high in “bonding” social capital, which is associated with a greater focus on kin relationships with a stronger social cohesion and stronger enforcement of social norms [68–70]. We also expect that AI/AN emerging adults who have personal networks with highly central members who identify as AI/AN will also be more likely to have higher connections to their cultural heritage.

Finally, we examine associations between individual characteristics of urban AI/AN emerging adults and feelings of cultural connectiveness and thoughts of historical loss. First, we hypothesize that demographic characteristics, such as age, gender, and socio-economic status, will be associated with these two outcomes. For example, older emerging adults who do not live in reservation/tribal areas may become more connected to their culture as they age and spend more time learning about their tribal heritage, including the history of losses suffered by the AI/AN population [71]. On the other hand, older emerging adults who do not live in reservation/tribal lands [72, 73] may become more removed from feeling connected to their heritage as they transition into adulthood and become more independent from their families. We also expect that socio-economic status may influence feelings of cultural connections. Those who come from families with economic resources may be able to afford involvement in cultural activities that require travel, such as visiting reservations or attending youth targeted cultural activities (e.g. the Gathering of Native Americans (GONA) [74]). On the other hand, higher socioeconomic status (SES) may increase the social interaction of AI/AN emerging adults with higher SES people, which may make them feel more aligned with the majority ethnic culture in the U.S. due to the network effect of homophily [75]. We also hypothesize that exposure to reservations/tribal lands and speaking a traditional language at home with families will be significantly associated with a greater connection between AI/AN emerging adults and their cultural heritage. In contrast, we hypothesize that the amount of time living in urban areas will be associated with lower cultural connection. We expect that gender and sexual/gender minority identity will be associated with cultural pride and thoughts of historical loss because of studies showing differences in ethnic identity between AI/AN male and female youth [76, 77] as well as higher experience of historical trauma by AI/AN sexual minority adults [78].

Overall, results will provide an important understanding of the social networks of urban AI/AN emerging adults currently lacking in the literature, which can help inform health interventions for AI/AN people. Urban AI/AN emerging adults have unique social influences, strengths and challenges that should be considered when developing a customized health intervention. Interventions that are tailored for AI/AN populations, are grounded in cultural traditions, promote social connections, cultural pride, and counter feelings of historical loss are necessary to counteract health disparities that are a result of inter-generational historical trauma [31].

## Data and Method

Participants (N = 150) are part of a randomized controlled trial, TACUNA (Traditions and Connections for Urban Native Americans) testing effects of two culturally appropriate interventions on alcohol and other drug (AOD) use and cultural connectedness [65]. Data collection for the current study occurred during the pandemic from December 2020 to October 2021; therefore, recruitment occurred online via social media across the U.S., and participants completed surveys online. Participants who responded to an online advertisement were directed to an online screener to determine eligibility. Eligibility criteria include: 1) age 18 to 25; 2) living in an urban area in any state in the U.S. that is not on a rancheria or a reservation); 3) self-identification as AI/AN; and 4) English speaking. The parent trial is specifically focused on prevention of opioid use disorder, and therefore focused on emerging adults who were not in need of treatment. Therefore, participants were also screened for the absence of an opioid use disorder with the Rapid Opioid Dependence Screener [79]. Those who completed the screener and were eligible were contacted by research staff consented to be part of the study. They were then asked to complete an online survey and randomized to receive either one virtual workshop or three virtual workshops and a Wellness Circle [65]. Data for this paper originate from the baseline survey prior to participation in workshops. The data analysis conducted for this study address the aims of a supplemental grant which funded additional analysis of the social network data. Participants received a $40 Amazon gift card upon survey completion. Procedures were approved by the RAND Human Subjects Protection Committee, including procedures for obtaining informed consent.

### Measures

#### Individiual characteristics.

Respondents reported their **age** in years and the **educational level of their mothers** (less than high school, high school, some college/AA degree, BA) as a proxy measure of socioeconomic status. In two separate questions, respondents reported the **percent of their life they had spent in urban areas and percent of their life spent on reservations or tribal lands.** They also reported on the **frequency they traveled to reservation/tribal lands** in the past year (never, 1–30 days, or > 30 days), **if they were born on reservation/tribal lands**, and if they **usually speak a tribal language** at home with families. Traditionally, Native American culture has respected sexual and gender diversity. The contemporary term “two-spirit” represents the respect for the co-mingling of feminine and masculine spirits in one person and previous studies have found an association between two-spirit identification and greater experience of Native-specific historical trauma [78]. To measure sexual gender minority identity broadly and to test for associations with cultural identity variables, respondents answered a series of questions to determine their **sexual gender minority (SGM)** identity. They reported their sex at birth, gender and transgender identification, sexual orientation, and gender(s) of past sexual partners. Participants could choose from a variety of answers to identify themselves demographically. For example, for what best describes their gender identity, participants could choose: a) female, b) male, c) gender fluid, d) something else, or e) prefer not to say. We defined respondents as sexual gender minority (SGM) individuals if they indicated any orientation other than “straight/heterosexual,” sex at birth being “something else” gender identity as “gender fluid” or “something else,” transgender identity, history of same-gender sex, or discordance between sex at birth and gender identity.

#### Social Network Measures.

To capture the expected diversity in social connections, we measured respondents’ social networks with a personal network (“egocentric”) survey instrument [66, 67]. This provided the raw data to produce measures of network composition (the types of people in the network) and network structure (the size and interconnections of this group of people). We asked respondents (“egos”) to name up to 15 people whom they talked with the most over the past three months (“alters”). Respondents were asked to list names with the following prompt:

The next set of questions will be about people you know. First, think about the people you have talked with the most over the past three months, either in person or over the phone, or by texting, emailing… things like that. Please type names of 15 people who are at least 18 years old. You will be asked questions about each of these people in the following screens. Do not enter full names. You can use their first names, nicknames, or some description that you will remember.

The count of these alter names entered by the participant is the egocentric **network size**. For each alter, respondents were asked a series of “name interpreter” questions. They were asked what their relationship was with each alter (Was the alter a family member, friend, romantic partner, etc.?). Based on these responses, we generated a measure of proportions of alters in each relationship type for each respondent, including **proportion “family”, “friend”, “romantic partner”, “co-worker”, “classmate”, “neighbor”, “teacher”, and “coach”.** We also asked respondents to classify each alter into an age category relative to their own age and generated a measure of proportion of alters who were **“older”, “around my age”,** or **“younger”** age. Respondents rated how close they were to each alter with a series of questions addressing different dimensions of relationship strength. Respondents reported the distance each alter lived from them (**same household, 0–5 miles, 5–15 miles, 15–50 miles, > 50 miles**) and the frequency of contact they had with the alter (**weekly, monthly, or less than monthly**). Respondents indicated if they received different types of support from each network alter, including **emotional support** or encouragement, **informational support** (advice), or **tangible support** (money, transportation, food, or other things).

Respondents were also asked to report the AI/AN identity of each alter. Respondents were asked if alters identify as AI/AN (do they “think of themselves as American Indian / Alaska Native”, yes/no). For those whom the respondent indicated that they do identify as AI/AN, they were asked if the alter does or does not **engage in AI/AN cultural/traditional activities** and if the alter ever **lived on a reservation or other tribal land**. For all alters, respondents indicated if they ever **discussed their own AI/AN identity** with the alter and how recently this discussion happened (**within the past 3 months, between 3–12 months, or over 12 months ago**).

To generate relationship data among the network alters named in each respondent’s personal network, we asked each respondent to evaluate if each unique pair of alters know each other. We generated a measure of **density** to evaluate the overall amount of connections in the network (i.e. the number of “edges”). Density is a ratio of the overall number of connections (the number of alter-alter pairs who know each other) to the maximum number of unique pairs of alters ((n*(*n*-1))/2 for a network the size of *n*). Density ranges from 0 (no connections) to 1 (everyone is connected). To measure connectivity of individual network members, we calculate *degree* for each network member, which is defined by the number of other network members that each alter knows. To measure the average connectivity of AI/AN alters across each personal network, we calculate the **average connectivity to AI/AN alters** by counting the number of edges connecting to AI/AN alters.

#### Dependent Variables.

We measured the connection between the respondent and their report of cultural pride and belonging and thoughts of historical loss. We assessed participants’ sense of **cultural pride and belonging** with the Multigroup Ethnic Identity Measure (MEIM) adapted for AI/AN heritage and used previously with a sample of AI/AN adolescents living outside of reservations/tribal lands [80] (α = .94). Respondents are asked the degree to which they agree with twelve statements such as, ‘I have a clear sense of my AI/AN identity and what it means to me’ on a scale from 1 = ‘strongly disagree’ to 5 = ‘strongly agree’ [81, 82]. We also assessed internalizations of the experiences of generational trauma for participants that can be psychologically distressing using the **Historical Loss (HL)** scale [83–85]. This measure was developed to measure the frequency with which Indigenous individuals contemplate losses to their culture, land, and people as a result of European colonization. Repeated thoughts about these generational experiences have been found to be psychologically distressing for Indigenous adolescents [83–85]. AI/AN young people who are SGM (“two-spirit”) have been found to have higher scores on historical loss contemplation than non-SGM AI/AN young people [78], likely due to the additional imposition of constricting European gender norms onto non-binary traditional gender expectations.

### Analyses

To address our first aim, we produced summary measures of network composition and structure for each respondent network and analyzed these measures across the sample with summary descriptive measures, including mean, standard deviations, min, and max. To summarize composition measures at the personal network level, we counted the number of network members with a specific characteristic and then divided this count by the total number of alters named by the respondent to produce a proportion for each network measure. We also calculated summary measures of network structure for the full sample based on the individual measures of each personal network. Finally, we present summary measures (means, standard deviations for continuous variables and percentages for categorical measures) of the characteristics of the respondents.

To address our second and third aims, we constructed two multiple ordinary-least squares (OLS) regression models to test associations between network characteristics and individual participant characteristics with each of our dependent variables (cultural pride and belonging and historical loss). These multivariate models were constructed to test for associations between measures of networks and individual characteristics while controlling for other variables in the model. To identify which variables to include in the multivariate model, we first tested a series of bivariate models that tested for significant associations between each candidate independent variable and each outcome variable. Each variable with a *p-value* < .10 was included in a multivariate model predicting each dependent variable. Because we asked respondents to name 15 alters, only a small number of respondents deviated from this instruction. Therefore, we include a dichotomous measure of egocentric network size (= 15 vs. <15) as a control in the multivariate model to account for any bias that may result from smaller networks. Because measures of network proportions ranged from 0 to 1, these measures were converted to deciles (multiplied by 10) to aid in interpretation of model results. All analyses were run using R 4.2.1. Regression analyses were run using the *glm* function of the R stats package.

## Results

### Descriptive Results

The 150 participants in the sample were mostly female (86%) and the average age was 21.8 (Table 1). Approximately two-thirds of the sample’s mothers had some education above High School with 36.7% having achieved a Bachelor’s degree. Respondents averaged 80.9% of their lives in urban areas and 22% of their lives on reservations / tribal lands, with only 14% having been born on a reservation or tribal land. Most respondents (48%) spent between 1–30 days traveling to reservations/tribal lands in the past year with 16% traveling more than 30 days and 36% not having traveled at all. Most respondents did not usually speak a tribal language at home (79.3%). Based on the combination of responses to questions about self-reported gender and sexual orientation, 48% reported SGM identity.

Table 2 presents network characteristics of the sample. Most respondents (87%) provided 15 names at the name generator prompt. The overall average number of alters named was 14.2 and the smallest network included only 3 alters. On average, the egocentric networks had a density of .45 with some networks having very low density (.067) and others having fully connected networks. Networks were composed mostly of friends and family members; on average, egocentric networks included 45.4% friends and 35.2% family members. Other types of network members named most frequently included romantic partners (5.9%), co-workers (6.6%), classmates (1.6%), neighbors (3.9%), teachers (7.4%), and coaches (7.4%). Respondents named an average of 59% alters whom they classified as “around my age”, 35.4% who were older, and 4.17% who were younger. Respondents had contact with an average of 58% of their alters at least once a week, 26% once a month or more (but less than weekly), and 13.6% less than once per month. Respondents received emotional support (74.2%) and information support (71.6%) from most of the alters they named. Most received tangible support from almost half of the members of their personal networks (44.3%). Less than half (40.1%) of alters named in the personal networks of the respondents identified as AI/AN and around a quarter (25.1%) engaged in traditional AI/AN activities. An average of 21.8% of the alters named by respondents had ever lived on a reservation or tribal lands. Respondents named an average of 21.7% of network members with whom they had never discussed their AI/AN identity. They discussed their AI/AN identity with around three-fourths of their networks, including over half (51%) within the past 3 months, 15.3% within the past year (but not the past 3 months), and 7.4% over 12 months ago. On average, there were 2.63 connections to AI/AN alters across all respondent personal networks.

**Figure 1:**
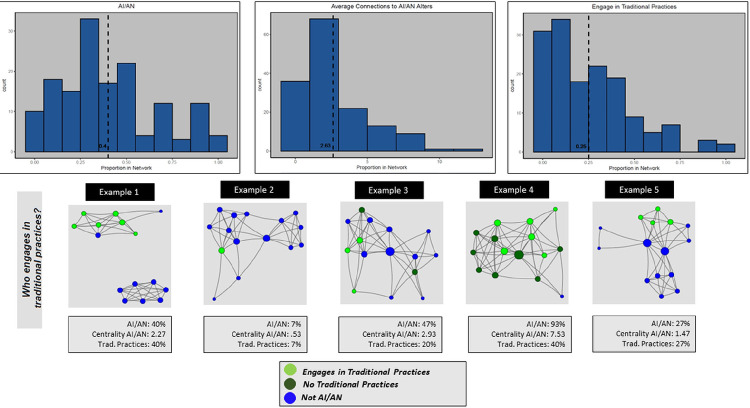
Distribution of AI/AN Discussion Partners and Example Personal Networks. Histograms showing distribution of three network measures: (left) Proportion of alters who identify as AI/AN; (center) Average connections to AI/AN alters; (right) Proportion of alters who engage in traditional practices. The figure also presents five example respondent egocentric networks illustrating the three measures. Each of the diagrams illustrate characteristics of alters with colored circles (“nodes”) and connections between alters as lines (“edges”). The nodes are placed on the plot with the R package “igraph” using the Fruchterman– Reingold spring embedding layout based on edges defined as alters who know each other. The light green nodes depict alters who identify as AI/AN and engage in traditional practices. Dark green nodes identify as AI/AN but do not engage in traditional practices and blue nodes do not identify as AI/AN. Node size represents the number of connections the node has to other nodes in the graph (“degree centrality”). Each example network diagram is illustrated with three measures: % alters who identify as AI/AN, average centrality of AI/AN alters, and % of alters who engage in traditional practices.

**Figure 2:**
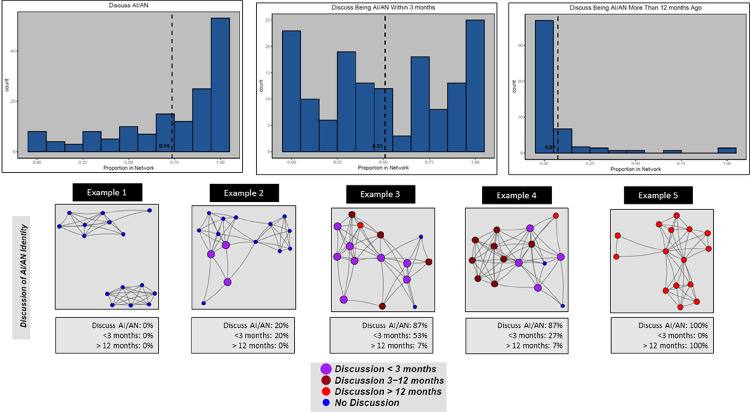
Distribution of AI/AN Status and Example Personal Networks. Histograms showing distribution of three network measures: (left) Proportion of alters with whom the respondent has discussed being AI/AN; (center) Proportion of alters with whom the respondent has discussed being AI/AN within the past 3 months; (right) Proportion of alters with whom the respondent has discussed being AI/AN more than 12 months ago. The figure also presents five example respondent egocentric networks illustrating the three measures. Each of the diagram illustrate characteristics of alters with colored circles (“nodes”) and connections between alters as lines (“edges”). The nodes are placed on the plot with the R package “igraph” using the Fruchterman–Reingold spring embedding layout based on edges defined as alters who know each other. Node size and color represent how recent the respondent has discussed being AI/AN with the alter with the largest nodes representing most recent (light purple nodes) and the smallest nodes representing those with whom the respondent has never discussed being AI/AN (small blue nodes).

Figure 1 illustrates the distribution of network characteristics across the sample, with histograms for proportion of alters identifying as AI/AN (left), average centrality of AI/AN alters (center), and the proportion of alters who engage in traditional practices (right). Each histogram displays the count of respondents having a range of proportions of alters with the focal characteristic. Figure 1 also provides five example personal network diagrams that depict individual respondent personal networks that illustrate the three measures. Similarly, Fig. 2 depicts three network variables with histograms and example diagrams on frequency and range of: proportion of alters with whom respondents ever discussed being AI/AN ever (left), within the past 3 months (center), and more than 12 months ago (right). The five network diagrams are for the same respondents’ personal networks as Fig. 1. The nodes in the Fig. 2 example diagram highlight different characteristics of alters with size and color. The largest nodes represent alters with whom the respondent discussed AI/AN identity with most recently. Purple nodes are those the respondent discussed being AI/AN most recently (light purple = < 3 months; dark purple = 3–6 months), red nodes are those the respondent discussed AI/AN identity over a year ago, and small blue nodes the alters with whom the respondent has never discussed AI/AN identity.

The five example diagrams in Figs. 1 and 2 highlight the diversity in network characteristics within our sample. For example, in diagram labeled Example 1 in Fig. 1, this individual has an average proportion of AI/AN alters with slightly below average centrality and above average proportion who engage in traditional practices. In Fig. 2, this respondent has not discussed being AI/AN with any of these alters. In contrast, the diagram labeled Example 4 in Fig. 1 has the highest proportion of AI/AN alters, including those who engage in traditional activities, and well above average number of connections to AI/AN alters. Figure 2 shows that Example 4 has also discussed being AI/AN with a higher than average proportion of alters, but lower than average proportion of discussions with alters within the past 3 months (and an average proportion more than 1 year ago).

The MEIM and Historical Loss items and scales (Tables 3 and 4) indicate high levels of cultural pride and belonging and contemplation of historical loss. The overall MEIM scores averaged 4.16, which is above an “Agree” response. The overall HL scale averaged 3.42, which represents an overall frequency of thoughts between “weekly” and “daily”. Each item fell into the range between “weekly” and “daily”.

### Bivariate Results

Table 5 presents results of bivariate tests of association between each of the network and individual measures and the MEIM / HL scale averages. Each row presents results of two different bivariate tests, one for each outcome variable. The table provides model estimates, 95% confidence intervals, and p-values. Results indicate that the following individual characteristics were significantly associated with MEIM scores: age, amount of time lived in reservations/tribal lands, days visiting tribal lands, being born on a reservation, and speaking a tribal language. Several network variables were also significantly associated with MEIM, including proportion of alters who were AI/AN, proportion who engage in traditional activities, proportion of alters whom the respondent discussed AI/AN in the past 3 months and over 1 year ago, and proportion of alters who have lived on reservations. The two variables measuring proportion of alters with whom the respondent discussed being AI/AN were significant in opposite directions: having more alters with whom the respondent had recent discussions was associated with higher MEIM scores whereas having higher proportions of alters with more distant (in time) conversations was associated with lower MEIM scores.

For HL scores, age and SGM status were the only individual characteristic measures significantly associated with HL scale scores. Most network variables were significantly associated with HL, including proportion of alters who were AI/AN, proportion who engage in traditional activities, proportion of alters whom the respondent discussed AI/AN in the past 3 months, 3–12 months, and over 1 year ago; and proportion of alters who have lived on reservations. The average connections to AI/AN alters was also significantly associated with higher scores on the HL scale.

### Multivariate Results

Table 6 provides estimates, 95% CIs, and p-values for each full multivariate model predicting MEIM and HL scores. Controlling for other factors, respondents with higher proportions of alters who engaged in traditional activities also had significantly higher MEIM scores. For each 10% increase in proportion of alters who engaged in traditional activities there was a predicted .092 increase in the MEIM score. Discussing AI/AN identity was also significantly associated with MEIM scores. An increase in 10% of alters with whom the respondent spoke about AI/AN identity increased MEIM scores by .058. For the model predicting HL scores, the only variable significantly associated with HL, controlling for other factors, was the proportion of alters with whom the respondent spoke to about being AI/AN recently. For each 10% increase in alters with whom the respondent spoke to about being AI/AN within the past 3 months, the HL score increased by .132. None of the individual characteristics were significantly associated with either the MEIM or the HL score, controlling for other individual and network measures.

## Discussion and Conclusions

The current study is the first to present a detailed analysis of the social networks of AI/AN emerging adults living in urban areas and provides empirical insights about the relationship between their social networks and their feelings of cultural pride and belonging as well as their thoughts of historical loss. These insights can inform the continued development of culturally-tailored health interventions for AI/AN populations. Understanding social networks among urban AI/AN emerging adults is critical to help inform ways to increase protective factors for this population. The continued health disparities experienced by AI/AN people in the U.S. indicate that existing evidence-based treatments are not addressing the needs of this population adequately [40]. Recent calls for innovative approaches to developing health interventions tailored for AI/AN populations suggest addressing social network factors that may be unique to AI/AN people [31, 63, 64].

Overall, findings demonstrate the common attributes as well as the range of network characteristics for this population. Participants’ networks were composed primarily of family and friends, mostly people their own age, and people with whom they had frequent contact. Respondents named, on average, high percentages of people who provide them with emotional support and advice (over two-thirds for each type of support). Also, nearly half of the people named, on average, were people with whom respondents had received money, transportation, food, or other tangible things. However, there was a broad range of network types with some networks composed of few of these types of members whereas others were dominated by one type of network member or another. The respondents’ networks were, on average 45% connected, but displayed a broad range of characteristics with some fully connected and others nearly completely disconnected.

Several network and individual characteristics suggest that AI/AN emerging adults living in urban areas have social worlds that are comprised of both urban and tribal area/reservation dynamics and interactions. Respondents reported living close to a quarter of their lives in reservation/tribal lands, with 14% reporting that they were born on reservation/tribal lands. Close to two-thirds had traveled to reservation/tribal lands in the past year, with 16% reporting having traveled there more than 31 days. On average, nearly 35% of network members named in personal network interviews lived over 50 miles away and nearly 22% had lived on reservation/tribal lands at some point during their lives. Taken together, findings show that AI/AN emerging adults living in urban areas have many social connections to reservations or tribal lands and most spend considerable time interacting with people there. An average of around 40% of networks included people with AI/AN identity and around 25% engage in traditional practices. On average, respondents named only around 22% of network members with whom they had never discussed their AI/AN identity and over half with whom they had discussed their identity with in the past 3 months. However, there was a broad range of network compositions in our sample, with some participants having no network members with whom they discuss their AI/AN identity and/or who identify as AI/AN and others having all AI/AN network members and discussing AI/AN identity with everyone in their networks.

In addition to descriptive findings, this study also demonstrates the important associations between social network characteristics and feelings of pride and belongingness for AI/AN emerging adults living in urban areas. Overall, the AI/AN emerging adult respondents in this study had high ratings on both the MEIM and the HL scales. This shows that, although they are geographically removed from tribal/reservation areas, they have a strong sense of cultural pride and agree that they feel they belong as part of their culture. They also think frequently about the traumas and losses that their people experience now and in the past. As anticipated, there was a significant association between measures on these constructs and how connected participants were with other AI/AN people in their social networks. Controlling for individual characteristics and other network measures, stronger feelings of cultural pride and belonging were associated with higher amounts of network connections with people who identified as AI/AN and engaged in traditional practices. Cultural pride and belonging was also significantly associated with larger proportions of network members with whom the respondent spoke to about AI/AN identity recently. Although several individual characteristics were significantly associated with cultural pride and belonging in tests of bivariate associations, none of these variables remained significant in the multivariate model. This finding underscores the strength of relationship between network factors and cultural pride and belongingness.

Only proportion of alters with whom the respondent engaged in discussions of being AI/AN in the past 3 months was significantly associated with thoughts of historical loss. It is not surprising that respondents who have frequent conversations about AI/AN identity with their social networks would have more thoughts of historically traumatic events that have impacted AI/AN people. Because thoughts of historical loss are associated with depressive symptoms among AI/AN adolescents [27], interventions that promote greater social connections and conversations between AI/AN people may increase recurring thoughts of historical loss, which could indirectly lead to an increase in depressive symptoms. However, other tribally led, culturally-tailored health interventions that provide education about AI/AN historical trauma have found only short-term increases in thoughts of historical loss among program participants [86], and by six-month post-test, thoughts of historical loss were no longer significantly increased. Studies have highlighted that interventions must also promote social connections and discussions with network members about AI/AN identity to help reduce negative health outcomes that may result from increased thoughts of historical loss [51, 85–89]. Thus, programs that include discussions of historical trauma need to have resources to support participants that may experience heightened mental health challenges as a result of increased thoughts of historical loss.

Although this study has many strengths and provides empirical evidence lacking in the literature on the social networks of AI/AN emerging adults living in urban areas, there are some limitations. First, the study is cross-sectional. Therefore, our findings cannot provide insights into causation and we are unable to determine if social network factors lead to cultural pride/belonging or thoughts of historical loss or if the network characteristics we observed are a result of characteristics of the respondents. Respondents for this study are participants in an ongoing RCT [65] that includes repeated measures of the same variables analyzed in this study. Analysis of longitudinal data from this study will enable more detailed tests of association over time as well as tests of the efficacy of the TACUNA program, which combines cultural programming, Motivational Interviewing, and personal network feedback, on AOD use prevention. Another limitation of this study is that our sample is mostly female and includes a high proportion of SGM respondents Further studies conducted among larger samples may offer an opportunity to further our understanding of the role of social networks and cultural identity on non-reservation AI/AN emerging adults; thus it is not representative. Further, although we controlled for these demographic characteristics in tests of associations with dependent variables and did not find any direct associations between them, it is possible that there are unknown biases that affect our findings.

In conclusion, our findings provide empirical data about social network characteristics of AI/AN emerging adults living in urban areas that have been lacking in the literature. This information is critical for developing tailored health interventions for this population to increase protective factors and help decrease the impact of inter-generational historical trauma. Findings highlight the importance of discussing social networks with urban AI/AN emerging adults to improve health outcomes through promotion of cultural strengths, pride, and belongingness while also recognizing the traumatic history of AI/AN people in order to promote healing. Although this study focuses on AI/AN people living in the United States, the findings are relevant for other displaced and historically traumatized populations around the world, such as international refugees or ethnic minority populations that are experiencing health disparities due to inter-generational trauma.

## Figures and Tables

**Table 1:** TACUNA sample Characteristics (n = 150)

Participant Characteristics	Mean(sd)	(%)

Age	21.8 (2.2)	

Sex at birth		14.0
Male		86.0
Female		0.0
Intersex/Other		

Mother’s education		8.7
Less than high school		21.3
High school		30.7
Some college/AA		36.7
Bachelor’s degree		2.7
Don’t know		

% of life spent in urban areas^a^	80.9 (28.1)	

% of life spent in reservations or tribal lands^a^	16.5 (27.0)	

Days traveled to reservation/tribal lands in past year		36
0 days		48
1 to 30 days		16
31 or more days		

Born on a reservation or tribal lands		14.0
Yes		86.0
No		

Usually speak tribal language at home with family		20.7
Yes		79.3
No		

Gender		12.0
Man		72.7
Woman		10.0
Gender fluid		4.7
Something else		0.7
Prefer not to say		

Sexual Orientation		48.7
Straight/heterosexual		2.0
Gay		4.7
Lesbian		30.7
Bisexual		4.7
Questioning		2.0
Asexual		5.3
Something else		2.0
Prefer not to say		

Identify as SGM		48.0

a Time spent in urban and reservation/tribal areas were asked in two separate questions. Respondents did not always provide answers that totaling 100%.

**Table 2:** Average Egocentric Characteristics (n = 150)

Variable Type	Social Network Variable	Mean (sd)	Range [min, max]
Network Structure			
	Size	14.2 (2.36)	[3, 15]
	Density	.45 (.20)	[.067, 1.00]
Network Composition (%)			
Relationship Type	Family	35.2 (22.5)	[0, 93]
	Friend	45.4 (23.4)	[0, 100]
	Romantic Partner	5.9 (5.6)	[0, 33]
	Co-worker	6.6 (11.7)	[0, 50]
	Classmate	1.6 (4.5)	[0, 27]
	Neighbor	3.9 (2.5)	[0, 27]
	Teacher	7.4 (3.1)	[0, 27]
	Coach	7.4 (3.1)	[0, 27]
Age	Same	59.0 (22.1)	[7, 100]
	Older	35.4 (20.7)	[0, 88]
	Younger	4.17 (7.1)	[0, 33]
Distance Live	Same household	12.2 (14.7)	[0, 100]
	Between 0 and 5 miles	16.5 (20.3)	[0, 100]
	Between 5 and 15 miles	19.4 (20.7)	[0, 100]
	Between 15 and 50 miles	12.9 (18.3)	[0, 100]
	Over 50 miles	34.5 (25.8)	[0, 100]
Frequency of Contact	Weekly	58.0 (23.2)	[7, 100]
	Monthly	26.5 (17.7)	[0, 73]
	Less than monthly	13.6 (16.5)	[0, 73]
Support	Receives emotional support	74.2 (25.8)	[0, 100]
	Receives tangible support	44.3 (29.4)	[0, 100]
	Receives information support	71.6 (26.6)	[0, 100]
AI/AN Identity	Identifies as AI/AN	40.1 (26.7)	[0, 100]
	Engages in AI/AN traditional practices	25.1 (23.3)	[0, 100]
	Lived on reservation or other tribal land	21.8 (25.5)	[0, 100]
Discussed AI/AN Identity	Never	21.7 (28.8)	[0, 100]
	Within past 3 months	51.0 (35.4)	[0, 100]
	Between 3 and 12 months ago	15.3 (24.4)	[0, 100]
	Over 12 months ago	7.4 (19.6)	[0, 100]
Network Centrality	Average degree to AI/AN alters	2.63 (2.35)	[0, 12.53]

**Table 3:** Average ratings of Cultural Pride and Belonging (MEIM)^a^

Item Text	Mean (sd)
I have spent time trying to find out more about my AI/AN identity	4.47 (0.87)
I am active in groups that include mostly members of my AI/AN group	3.31 (1.36)
I have a clear sense of my AI/AN identity and what it means for me	3.97 (1.06)
I think a lot about how my life will be affected by my AI/AN identity	4.08 (1.02)
I am happy that I am a member of the AI/AN tribal group I belong to	4.68 (0.66)
I have a strong sense of belonging to my AI/AN tribal group	3.87 (1.14)
I understand pretty well what my AI/AN identity means to me	4.14 (0.96)
In order to learn more about my AI/AN identity, I have often talked to other people	4.16 (0.92)
I have a lot of pride in my AI/AN identity	4.70 (0.62)
I participate in cultural practices of my own AI/AN tribal group	3.80 (1.14)
I feel a strong attachment towards my AI/AN tribal group	4.26 (0.92)
I feel good about my cultural background	4.46 (0.86)
Overall Scale Average:	4.16 (0.66)

a 5-point scale from Response of “Strongly Disagree” = 1 to “Strongly Agree” = 5

**Table 4:** Average ratings of Historical Loss scale (HL)^a^

	Mean (sd)
The loss of our land	3.52 (1.22)
The loss of our language	3.51 (1.22)
Losing our traditional spiritual ways	3.45 (1.25)
The loss of our family ties because of boarding/residential schools	3.05 (1.50)
The loss of families from the reservation/reserve/village to government relocation	3.11 (1.43)
The loss of self-respect from poor treatment by government officials	3.41 (1.40)
The loss of trust in whites from broken treaties	3.53 (1.37)
Losing our culture	3.74 (1.24)
The losses from the effects of alcoholism on our people	3.51 (1.35)
Loss of our people through early death	3.33 (1.40)
Overall Scale Average:	3.42 (1.11)

a 6-point scale “Never” = 0, “Several Times a Day” = 5

**Table 5:** Bivariate OLS Regression models predicting MEIM and HL (n = 150)

	MEIM			Historical Loss	
Variable	Est	95% CI	p-val	Est	95% CI	p-val
Age	.003	(−.029,.036)	.84	.058	(.000,.117)	.05
Mother education - BA (vs. less than BA)	.182	(.033,.331)	.02	−.224	(−.495,.047)	.11
Female gender	.087	(−.111,.285)	.39	.073	(−.286,.431)	.69
% of life lived in urban area	−.002	(−.004,.000)	.12	.002	(−.002,.006)	.40
% of life lived in reservations or tribal lands	.003	(.001,.005)	.01	−.002	(−.006,.002)	.31
Days visiting reservations or tribal lands	.267	(.166,.368)	<.01	.097	(−.092,.287)	.31
Born on reservation or tribal lands	.249	(.064,.435)	.01	−.106	(−.445,.234)	.54
Usually speak tribal language at home with family	.309	(.142,.476)	<.01	.245	(−.062,.552)	.12
Sexual / Gender Minority	−.114	(−.256, .028)	.12	.245	(.073, .583)	.01
Proportion Alters who think of themselves as AI	.085	(.048,.122)	<.01	.105	(.040,.170)	<.01
Proportion alters who engage in traditional activities	.122	(.081 ,.163)	<.01	.13	(.056,.204)	<.01
Proportion alters respondent has discussed being AI/An < 3 months	.076	(.049,.104)	<.01	.139	(.083,.290)	<.01
Proportion alters respondent has discussed being AI/An 3–12 months	−.029	(−.072,.014)	.19	−.111	(−.067,.180)	<.01
Proportion alters respondent has discussed being AI/An > 12 months	−.090	(−.142,−.038)	.01	−.123	(−.139,.207)	.01
Proportion alters who lived on reservation	.079	(.040,.119)	<.01	.065	(−.005,.134)	.07
Density of Egocentric Network	−.287	(−.813,.238)	.29	−.52	(−1.407,.367)	.25
Average # of Connections to AI Alters	.064	(−.008,.208)	.38	.236	(−.024,.496)	.08

**Table 6:** Multivariate OLS Regression Models predicting MEIM and HL (n = 150)

	MEIM			Historical Loss	
Independent Variables*	Est	95% CI	p-val	Est	95% CI	P-val
Age	--	--	--	.031	(−.041, .103)	.40
Mother education - BA (vs. less than BA)	.090	(−.096, .277)	.34	--	--	--
% of life lived in reservations or tribal lands	.000	(−.004, .004)	.94	--	--	--
Days visiting reservations or tribal lands	.121	(−.024, .266)	.10	--	--	--
Born on reservation or tribal lands	.129	(−.153, .410)	.37	--	--	--
Usually speak tribal language at home with family	.195	(−.053, .443)	.13	--	--	--
Sexual / Gender Minority	--	--	--	.099	(−.216,.415)	.54
Proportion Alters who think of themselves as AI	.002	(−.060, .065)	.94	.038	(−.129,.205)	.66
Proportion alters who engage in traditional activities	.092	(.029, .154)	<.001	.054	(−.061 ,.169)	.36
Proportion alters respondent has discussed being AI/AN < 3 months	.058	(.030, .086)	<.01	.132	(.076,.188)	.00
Proportion alters respondent has discussed being AI/AN 3 – 12 months	--	--	--	−.012	(−.088,.063)	.75
Proportion alters respondent has discussed being AI/AN > 12 months	−.405	(−.896, .086)	.11	−.019	(−.109,.071)	.68
Proportion alters who ever lived on reservation	−.118	(−.753,.517)	.33	−.332	(−1.265,.602)	.49
Average # of Connections to AI/AN Alters	--	--	--	.055	(−.078,.188)	.44
						

* Controlling for egocentric network size = 15 vs. <15

## Data Availability

The data underlying this article will be shared in a repository that is currently in progress. Contact the first author, Dr. David Kennedy, or the PIs of the project grants, Dr. Elizabeth J. D’Amico and Dr. Daniel L. Dickerson, with any queries.
